# Utilization of modern contraceptives and associated factors among street women in Dire Dawa, Eastern Ethiopia: a mixed study

**DOI:** 10.1186/s12978-021-01263-z

**Published:** 2021-10-26

**Authors:** Alemu Guta, Bezhabh Amsalu, Tadesse Weldamanuel, Alekaw Sema, Legesse Abera, Bekele Simegn Demissie, Yalelet Belay

**Affiliations:** 1 Department of Midwifery, College of Medicine and Health Science, Dire Dawa, Ethiopia; 2 Department of Public Health, St. Lideta College of Health Sciences, Addis Ababa, Ethiopia

**Keywords:** Modern contraceptives, Reproductive-age, Street women, Dire Dawa

## Abstract

**Introduction:**

Unwanted pregnancy and sexually transmitted diseases are the major problems in street women because of the non-utilization of modern contraceptives. To the best of our knowledge, no studies have assessed the utilization of modern contraceptives and associated factors among street women in the study area. Therefore, this study aimed to determine the utilization of modern contraceptives and its associated factors among street women.

**Methods:**

A community-based cross-sectional study using mixed methods was conducted from February 16th to April 10, 2021, among all censuses and 615 reproductive-aged street women. Face-to-face and in-depth interviews were employed to generate quantitative and qualitative data, respectively. Binary logistic regression was used to analyze the association between modern contraceptive utilization and independent variables with statistical significance set at p < 0.05. Qualitative data were analyzed using a thematic approach.

**Results:**

Approximately half 279 (50.3%) (95% CI: 46.3%, 54.4%) street women currently used modern contraceptives. Factors significantly associated were women aged 25–34 years [AOR = 5.5, 95% CI: 1.2–24], distance from a nearby health facility within 30 min [AOR = 9.2, 95% CI: 1.6–51], getting advice from health professionals [AOR = 14.3; 95% CI = 5.3–38.4], discussed with their sexual partners about the utilization of modern contraceptives [AOR = 6.2, 95% CI: 2.4–16.5], a history of pregnancy [AOR = 2.7, 95% CI: 1.2–6], the desire to have a child after two years [AOR = 2.2, 95% CI: 1.1–4.7], and women who faced rape in street life [AOR = 5.4; 95% CI = 1.9–15.3]. Fear of side effects, misperceptions, and the desire to have a child are the main identified themes for the key barriers to using modern contraceptives.

**Conclusion:**

The proportion of street women currently using modern contraceptives was low. Age, distance from the health facility, discussion with health professionals, discussion with sexual partners, history of pregnancy, desire time to have a child in the future, and history of rape were factors significantly associated with the use of modern contraceptives. Most of the participants’ reasons for the lack of use of modern contraceptives were fear of its side effects.

## Plain language summary

Street women are a segment of the population in absolute poverty, which they suffer from the major problems of unwanted pregnancy and sexually transmitted diseases. Besides this issue, its consequences necessitate the use of modern contraceptives. This study aimed to determine the utilization and associated factors, and explore the key barriers to modern contraceptive utilization among street women in Dire Dawa.

In this study, a community-based cross-sectional study design was conducted among all censuses and 615 reproductive-aged street women from February 16th to April 10, 2021. Face-to-face and in-depth interviews were employed to generate data for both methods. Data were analyzed using the SPSS software version 25 and a thematic approach for quantitative and qualitative, respectively.

The findings of this study showed that the proportion of street women who currently used modern contraceptives was 279 (50.3%). Factors significantly associated were age, distance from the health facility, discussion with health professionals, discussion with sexual partners, history of pregnancy, desire time to have a child, and history of rape. The key barriers to using modern contraceptives are fear of side effects, misperceptions, and the desire to have a child.

In conclusion: this study demonstrated that the proportion of street women currently using modern contraceptives was low. Health professionals and health extension workers should give health education that integrates well-organized street women’s groups and community health extension workers that may help reduce women's fear of potential side effects. To correct the misperceptions and myths about modern contraceptives too.

## Introduction

Family planning refers to a conscious effort by a couple to limit or space the number of children they have through the use of contraceptive methods. Modern contraceptive methods include the pill, injectable, male and female condoms, emergency contraception, implants, intrauterine contraceptive device (IUCD), female and male sterilization, standard day method (SDM), and lactational amenorrhea method (LAM) [1]. Worldwide, there are 1.9 billion women in the reproductive age group (15–49 years) in 2019, 1.1 billion need family planning; of which 842 million are using contraceptive methods, and 270 million have an unmet need for contraception [2, 3]. In Ethiopia, 41% of currently married women use modern contraceptive methods, and 1% use natural methods [1].

The prevalence of modern contraceptives among women of reproductive age has increased worldwide between 2000 and 2019 by 2.1% (55 to 57.1%) [2]. Reasons for this slow increase include a limited choice of methods; limited access to services, particularly among young, poorer, and unmarried people; fear or experience of side-effects; cultural or religious opposition; poor quality of services; users' and providers' bias against some methods; and gender-based barriers to accessing services [2, 4]. Target 3.7 of the Sustainable Development Goals (SDGs) calls on countries “by 2030, to ensure universal access to sexual and reproductive health-care services, including for family planning, trends in contraceptive prevalence and need for family planning satisfied with modern methods indicate where increased investments and commitments by governments and international organizations are needed for the realization of reproductive rights for all people, and to help fulfill the pledge of the 2030 Agenda for Sustainable Development that “no one will be left behind” [3].

The World Health Organization has clarified that modern contraceptive methods are essential for preventing pregnancy-related health risks in women, reducing infant mortality, helping to prevent HIV/AIDS, empowering people and enhancing education, reducing adolescent pregnancies, and slow population growth [5, 6].

Sexual and reproductive health problems are responsible for one-third of the health issues of women of reproductive age (15–49 years). Unsafe sex is a major risk factor–particularly among women in developing countries. Therefore, it is important to obtain services for 222 million women who do not receive the contraception services they need [7].

Street women are women who make their lives on street life by begging, sleeping in streets, or on roadsides. Those who had no formal homes (homeless) and sleep on streets, verandas, balconies, etc. at night were classified as "on-street" while those who have houses to go for sleep at night while making their lives on the street life were termed as "off-street" [8]. They are the most marginalized, neglected part of society, with very negligible access to health care, including modern contraceptives, and a lack of awareness of health services. The circumstances in which they live and work increase their vulnerability to sexual exploitation and abuse and put them at a higher risk of unwanted pregnancies, sexually transmitted infections, and HIV/AIDS [9, 10]. In addition, research has revealed that women living on the streets are less likely to benefit from basic reproductive health services, including modern contraceptives as living in the poorest segment of society [11]. To address this problem, most homeless females do not have access to information regarding sexual health and safety. Homeless mothers showed a profound lack of knowledge or interest in birth control and reproductive health, and 50% of them did not believe that birth control was important [12].

Similarly, studies have shown that unwanted pregnancy and sexually transmitted diseases (STDs) are the major problems in street women because they do not use modern contraceptives [8, 13–15].

Street women are a segment of the population in absolute poverty. Around half of them are sexually active and have a history of pregnancy, even more than one pregnancy after they come into street life. One-third of street residents face sexual assaults in their street life [8]. Unwanted pregnancy and its consequences necessitate the use of modern contraceptives to control birth. STDs and unwanted pregnancies besides homelessness have negative consequences for the women themselves, their children, siblings, and societies [16].

Few studies have focused on the utilization of modern contraceptives among street women in the country with only a quantitative method [8, 17–19], and no recent comprehensive data regarding this in the study area in particular. In addition, as the findings of these previous studies varied, to obtain evidence-based epidemiological data in our study area, these studies only used quantitative data collection methods, unlike our study that used both quantitative and qualitative methods to explore the key barriers of modern contraceptive utilization by street women. Therefore, this study aimed to determine the utilization and associated factors, and further explore the key barriers to modern contraceptive utilization among street women in Dire Dawa.

## Methods and materials

### Study area and period

The study was conducted in Dire Dawa City from February 16th to April 10, 2021. Dire Dawa City is 515 km from Addis Ababa to the East. According to 2019 population projections, 313,000 people (63%) live in the City of Dire Dawa and 180,000 people (37%) live in rural areas [20]. The total fertility rate is 3.1 for women of reproductive age (15–49 years) [21]. In terms of the distribution of public health facilities, the city has 2 public hospitals, 8 health centers, 5 higher clinics, 12 medium clinics, and one FGAE. The administration has 9 urban Kebeles.

### Study design and population

Community-based mixed (quantitative and qualitative) studies were also conducted. All reproductive-aged street women who lived in Dire Dawa City were included, while pregnant and critically ill street women were excluded.

### Sampling and sampling procedures

All Census reproductive-aged (15–49 years) street women in Dire Dawa City during the data collection period. We conducted a pre-phase assessment and estimated the number of reproductive-aged street women, with 667 reproductive-aged street women. Of these, we excluded 4 critically ill and 48 pregnant women. Finally, 615 reproductive-aged street women were interviewed with a response rate of 90.24%. Fourteen (14) street women were included in the supplementary qualitative method based on the saturation of the data.

### Sampling techniques

After the census of street women in demarcated sites in nine kebeles, 667 women of childbearing age lived in the streets. The demarcated sites in each Kebele include main roadsides, isolated slum areas (bridge sides), around taxi/bus stations, traffic lights sides, road junctions, around Churches and/or Mosques, and streets where street women usually reside and/or sleep. Finally, a census survey of street women of reproductive age who fulfilled the eligibility criteria was included. Since street women are mobile and have no specific addresses, the probability of being interviewed by street women at other sites is high. To avoid interviewing more than once, women were asked whether they had previously been interviewed for modern contraceptive awareness and utilization within the period of data collection. For the qualitative method: purposive sampling techniques were used to select the interviewees.

### Operational definitions

We define street women as women who make their lives on street life by begging, sleeping in streets, or on roadsides. Those who had no formal homes (homeless) and sleep on streets, verandas, balconies, etc. at night were classified as "on-street" while those who have houses to go for sleep at night while making their lives on the street life were termed as "off-street" [8]. In this document, we frequently used interchangeably for both street women and beggar women to convey the same meaning.

Street women’s awareness of modern contraceptives was assessed using simple knowledge questions about modern contraceptives (if they have ever heard, able to mention methods, know the importance, source of the information, and site to get MCs). Modern contraceptive ever user: A woman who has ever used any of the modern contraceptive methods [17]. Modern contraceptive current users: women using modern contraceptive methods at the time of the survey [17, 18].

### Data collection instrument and measurements

Data were collected through face-to-face interviews using piloted and validated surveys, which were adapted from the literature. The tool included different sections, such as sociodemographic characteristics, awareness and utilization of street women toward modern contraceptives, and reproductive health and sexual exposure-related factors [8, 8, 11, 17–19, 22]. First, we prepared the tool in English and then translated it into the local language and back to English. Nine trained health professionals collected the data, and three supervisors and investigators supervised the data collection process. We collected data at the demarcated sites where more street women live, and the data collection times were Monday to Sunday, especially in the morning and afternoon.

Open-ended questions were used through in-depth interviews to collect the qualitative data. During the interviews, the dialog was recorded using a tape recorder, and field notes were recorded using a notebook and pen/pencil. Two trained health professionals collected the data. After the note taker and the interviewer introduced themselves, the purpose of the study and confidentiality of the data was explained to the participants, and the probing questions were forwarded to the participants. The in-depth interview took approximately 30 min to complete. Finally, the investigators transcribed the collected data in the full text on the same day.

### Data quality assurance

Data quality was assured by senior researchers and the adapted questionnaire was checked and revised. The training was provided for data collectors for a 2-days emphasis on the tool, the art of interviewing especially to address the sensitive question by tactic full manners, ethical issues, and the significance of the study, as well as how to approach the participants. During the pre-assessment phase, we conducted a pre-test on 20 women of data collection tools, and necessary corrections on the instrument were incorporated. Each data collector checked the questionnaires for completeness before visiting the participants. The investigators checked the data overnight. Then, necessary feedback was offered to the data collectors every morning.

The recorded data were transcribed on the same day to incorporate all the women's information, including their facial expressions, and only two interviews were recorded per day. Field notes were taken side by side with a tape recorder and expanded within 24 h to use as a backup in cases where the records failed. We checked the field notes and transcripts and recorded the interviews each day for errors to correct the timing. The interviews were then transcribed carefully, and data coding and thematic analyses were performed.

### Data processing and analysis

The data were checked for completeness and consistency. Finally, the data were entered into Epi-data 3.1 and exported to SPSS version 25.0. Frequency and percentages were used to illustrate the study findings in the tables and text. We analyzed the association between the outcome and independent variables using a binary logistic regression model. Variables with a p < 0.2 in the bivariate analysis were used for multivariable logistic regression analysis. Hosmer and Lemeshow's goodness-of-fit test was used to assess whether they fulfilled the necessary assumptions. We measured the direction and strength of the statistical associations using odds ratios with 95% confidence intervals (CIs). AOR with 95% confidence intervals (CIs) at p < 0.05 was considered as a statistically significant association with modern contraceptive utilization.

Qualitative data were translated and analyzed using a thematic approach with ATLAS.ti 8. We developed themes after reflexive reading of the translations. All authors cross-validated emerging themes.

## Result

### Sociodemographic characteristics of street women

Of the total, 555 street reproductive-aged women were interviewed, resulting in a response rate of 90.24%. The mean age of the women was 28.52 years (SD ± 7.47). Most women were aged 25–34 years (47%). The majority, 467 (84.1%) and 456 (82.2%) women were Oromo and Muslim followers, respectively. Regarding the educational status of women, 380 (68.5%) were unable to read and write. The mean duration of street women who stayed in street life was 5.98 years (SD ± 5.32) (Table 1).

**Table 1:** Sociodemographic characteristics of street women in Dire Dawa (n = 555)

Variables		Frequency (N)		Percentage (%)
*Age of women (years)*				
15–24		166		29.9
25–34		261		47.0
35–49		128		23.1
*Religion*				
Orthodox		82		14.8
Muslim		456		82.2
Others***		17		3
*Ethnicity*				
Oromo		467		84.1
Amhara		64		11.5
Somale		17		3.1
Others**		7		1.3
*Education status of women*				
Cannot read and write		380		68.5
Read and write only		79		14.2
Elementary (Grade 1–8th)		91		16.4
High school (Grade 9–12th)		5		0.9
*Residence before street life*				
Urban		225		40.5
Rural		330		59.5
*Sexual partners*				
Yes		352		63.4
No		203		36.6
*Sleeping at night?*				
On-street		460		82.9
Off-street		95		17.1
*Duration of street life (years)*				
0.5–5		345		62.2
6–10		140		25.2
11–15		39		7.0
16–20		19		3.4
> 20		12		2.2
*Daily income*				
< = 30 birr		334		60.2
> 30 birr		221		39.8
*Family size*				
1		111		20.0
2–3		163		29.4
> 3		281		50.6
*Have a dependent family member?*				
Yes		347		62.5
No		208		37.5
*Disability*				
Yes		83		15.0
No		472		85.0
*Types of disability (N* = *83)*				
Blindness		10		12.0
Problem on hand or leg		29		34.9
Non-specified internal problem		23		27.7
Mental health problem		3		3.6
Difficulty hearing		11		13.3
Others***		7		8.4
*Knows the place where the health service is found?*				
Yes		540		97.3
No		15		2.7
*The time it will take to arrive at the nearby health facility on foot (N* = *540)*				
< = 30 min		492		91.1
> 30 min		48		8.9

***Protestant, catholic, pagan, **Tigray, Gurage, **HIV/AIDS, epilepsy

### Awareness of street women about modern contraceptives

Most 516 (93%) women said they had information (heard) about modern contraceptives, and injectable 455 (88.2%) was the most commonly mentioned. More than half 260 (50.4%) of their sources of information were health professionals. The majority of the participants, 465 (90.3%) agreed that modern contraceptives had their advantages. More than half 363 (70.4%) of the street women were discussed with health professionals about modern contraceptives (Table 2).

**Table 2:** Awareness of street women towards modern contraceptive methods in Dire Dawa, Ethiopia, 2021 (n = 555)

Variables	Frequency	Percent%
*Has information about modern contraceptives*		
Yes	516	93
No	39	7
*Modern contraceptives mentioned by respondents (N* = *516)*		
Pills	303	58.7
Injectable	455	88.2
Condoms	138	26.7
Implants	407	78.9
IUCD	99	19.2
Female sterilization	8	1.6
Vasectomy	4	0.8
*Source of information about modern contraceptives (N* = *516)*		
Family	12	2.3
Friends	249	48.3
Health professional	260	50.4
Media	25	4.8
Others*	5	1
*Know the advantages of modern contraceptives mentioned? (N* = *516)*		
Yes	465	90.3
No	50	9.7
*Advantages of modern contraceptives mentioned (N* = *465)*		
Spacing births	348	74.4
Limit births	45	9.6
Prevent unwanted pregnancy	139	29.7
Others*	4	0.9
*Know the place to get MCs (N* = *516)*		
Yes	502	97.5
No	14	2.5
*A place to get modern contraceptives (N* = *502)*		
Pharmacy	54	10.8
Health centers	466	92.8
Hospitals	74	14.7
Others*	22	4.4
*Advice from health professionals about modern contraceptives (N* = *516)*		
Yes	363	70.35
No	153	29.65
*Discuss modern contraceptive utilization with sexual partner (N* = *516)*		
Yes	170	33
No	346	67

*Reading; *Family Guidance Association of Ethiopia

### Utilization of modern contraceptive methods among street women

Of the total women, 356 (64.1%) in this study used one of the modern contraceptives while 279 (50.3%) (95% CI: 46.3%, 54.4%) were currently using modern contraceptives.

Among the current modern contraceptive users, 159 (57%) used implants followed by injectable 99 (35.5%, pills, condoms, and IUCD. Most of the 238 (85.3%) participants took it from health centers followed by the hospital, FGAE, and pharmacy where 16 (5.7%), 14 (5%), and 11 (3.9%), respectively. Regarding the reason for not using modern contraceptives currently were fear of perceived side effects 103 (37.3%) followed by being sexually not active 102 (36.96%), lack of knowledge 39 (14.1%), lack of interest 27 (9.78%), lack of access 4 (1.4%), and only one woman did not have a reason. Among those who were not currently using contraceptives, 191 (69.2%) did not plan to use modern contraceptives in the future.

### Sexual behaviors and reproductive health of street women.

Of the total, 333 (60%) practiced sexual activity in the six months prior to data collection. Over sixty percent, 339 (61.1%) of the participants had a history of pregnancy after engaging in street life. Ninety-nine (17.8%) reported that they had a history of rape while living on the streets (Table 3).

**Table 3:** Reproductive health-related and sexual exposure factors of street women in Dire Dawa, Ethiopia, 2021 (n = 555)

Variable	Frequency	Percent %	
*Sexually active in the six months*			
Yes	333	60	
No	222	40	
*History of pregnancy after joining street life*			
Yes	339		61.1
No	216		38.9
*Number of pregnancies in street life (N* = *339)*			
1	133		39.2
2	108		31.9
> 2	98		28.9
*Is the pregnancy is planned? (N* = *339)*			
Yes	245		72.3
No	94		27.7
*Number of alive children*			
0	140	25.2	
1–2	224	40.4	
> 2	191	34.4	
*History of child loss*			
Yes	157	29.5	
No	376	70.5	
*History of termination of pregnancy after joining street (N* = *339)*			
Yes	135	39.8	
No	204	60.2	
*Desire to have a child in the future?*			
Yes	279	50.3	
No	276	49.7	
*Time of a plan to have a child in the future (N* = *279)*			
Within 2 years	160	57.3	
After 2 years	119	42.7	
*History of rape after joining street*			
Yes	99	17.8	
No	456	82.2	
*Number of rape (N* = *102)*			
One time	35	35.4	
Two and above times	64	64.6	

### Factors associated with utilization of modern contraceptives among street women

Participants aged 25–34 years were 5.5 times [AOR = 5.5, 95% CI: 1.2–24] more likely to utilize modern contraceptives than those aged 15–24 years. Women who arrived at a nearby health facility on foot within 30 min were 9.2 times more likely to utilize modern contraceptives than those who took more than 30 min [AOR = 9.2, 95% CI: 1.6–51]. Women who received advice from health professionals about modern contraceptives were 14.3 times more likely to utilize modern contraceptives than their counterparts [AOR = 14.3; 95% CI = 5.3–38.4]. Women who discussed the utilization of modern contraceptives with their sexual partners were 6.2 times more likely to utilize MCs currently as compared to their counterparts [AOR = 6.2, 95% CI: 2.4–16.5].

Street women who had a history of pregnancy after joining the street were 2.7 times [AOR = 2.7, 95% CI: 1.2–6] more likely to utilize MCs as compared to their counterparts. Women who desire to have a child after two years were 2.2 times [AOR = 2.2, 95% CI: 1.1–4.7] more likely to utilize modern contraceptives currently as compared to those women who have a child within two years. Women who faced rape in street life were 5.4 times [AOR = 5.4; 95% CI = 1.9–15.3] more likely to utilize modern contraceptives currently as compared to their counterparts (Table 4).

**Table 4:** Factors associated with utilization of street women toward modern contraceptives in Dire Dawa, Ethiopia, 2021

Variables	Utilization of MCs (N = 555)		COR (95% CI)	AOR (95% CI)	P-value
	Yes (N = 279)	No (N = 276)			
*Age of women (year)*					
15–24	87	79	1	1	
25–34	158	103	3 (1.8–5)	5.5 (1.2–24)	**0.024**
35–49	34	94	0.72 ( 0.5–1)	1.8 (0.7–4.6)	0.225
*Educational status*					
No formal education	223	236	1	1	
Elementary and above	56	40	1.5 (0.95–2.3)	0.6 (0.2–1.8)	0. 364
*Sexual partner*					
Yes	228	124	5.5 (3.7–8)	1 (0.45–2.4)	0.919
No	51	152	1	1	
*Residence before joining street*					
Urban	134	91	1.9 (1.3–2.65)	1.35 (0.6–2.8)	0.440
Rural	145	185	1	1	
*Sleeping at night*					
On-street	242	218	1.74 (1.1–2.7)	1.4 (0.6–3.6)	0.400
Off-street	37	58	1	1	
*The time it will take to arrive at the nearby health facility on foot*					
< = 30 min	267	225	4 (2–8)	9.2 (1.6–51)	**0.011**
> 30 min	11	37	1	1	
*Number of modern contraceptives mentioned*					
1–2	103	110	1	1	
3–4	144	117	2.6 (1.2–5.5)	3.4 (0.5–21)	0.187
> = 5	32	10	3.4 (1.6–7.3)	4 (0.6–28.6)	0.146
*Advice from health professionals about modern contraceptive*					
Yes	267	96	32.4 (17–61)	14.3 (5.3–38.4)	**0.000**
No	12	140	1	1	
*Discussion with sexual partners about the utilization of MCs*					
Yes	143	27	8 (5–12.9)	6.2 (2.4–16.5)	**0.000**
No	136	209	1	1	
*History of pregnancy after joining street*					
Yes	204	135	2.8 (2–4)	2.7 (1.2–6)	**0.015**
No	75	141	1	1	
*Desire time to have a child in the future*					
Within 2 years	80	80	1	1	
After 2 years	77	42	1.8 (1.1–2.9)	2.2 (1.1–4.7)	**0.038**
*History of rape*					
Yes	78	21	4.7 (2.8–7.8)	5.4 (1.9–15.3)	**0.002**
No	201	255	1	1	

*Statistically significant at p-value < 0.05 in multivariate logistic regression analysis

### Qualitative result

Most of the participants had no formal education and their ages were between and 25–34 years old. Regarding their duration of street life, more than half of them reported that they were on the street for 5 years and above. Three thematic categories emerged from the collected data: fear of side effects, misperceptions about modern contraceptives, and desire to have a child.

### Fear of side effects

Most of the participants’ reasons for the lack of use of modern contraception were fear of its side effects, especially after using implants and injectable methods, such as weight loss, severe headache, irregular bleeding, behavioral changes such as mood swings, and anger.

A 30-year-old woman of a mother of 2 children who had stayed 5 years after joining street life epitomized this view: “I discontinued Injectable (Limmoon Kan kennamu) after 3-term of utilization because it changed my face color [darkened skin], and stopped my menses [amenorrhea]… I shifted it to Implanon, and I also discontinued it because I experienced severe headache, weight loss, and behavioral changes like angry (I threw away my bay and run)”. (Participant 7).

### Misperceptions about modern contraceptives:

Street women overestimate the side effects of modern contraceptive methods based on hearsay from their friends and relatives about the methods. They perceived it to cause infertility; it is not used by poor women (street women) because it needs highly nutritional food (like milk, meat), and even kills women on the street. Negative anecdotes of modern contraceptive health issues have decreased when using contraceptive methods. In particular, they considered the implants and injectables to be bad.

A 20-years-old woman of 2 children, who stayed one and half years on the street said: “I do not use any methods of modern contraceptives because they are dangerous and can disappear in our body and it leads infertility…….” (Participant 10).

Another 25-year-old woman added: “I do not use modern contraceptives because as I heard from my friend she experienced behavioral change [manic mood] after she had used Implanon (she hates her baby).” (Participant 9).

### Desire to have a child

Some street women do not use or stop modern contraceptive methods because of their husband's pressure to have a child. Similarly, in the other case, some of the street women had the desire to have a child by themselves to continue their offspring.

A 30-years-old woman who stayed 7 years on street said that "I stopped Dipo (Limmoon Kan kennamu) after one year of utilization, because of my husband's pressure on me to have a child, and I gave birth one month ago [before the survey]”. (Participant 2).

Another 30-year-old woman of one child said that “I discontinued Implanon because I desired to have a child”. (Participant 12).

## Discussion

In this study, 279 (50.3%) street women were currently using modern contraceptives. This prevalence is higher than that reported in Chicago, USA, which showed that 16 women (30%) [23]. This difference might be because of the very small sample size in the previous study, and the elapsed study period. Similarly, this finding is higher than that of studies conducted in Ethiopia such as North-West (Bahir Dar and Gondar) in 2012, which was 70 (34.3%), Amhara Regional State Zonal Towns in 2020 which was 235 (38.9%), Bahir Dar 2020 was 73 (31.1%), SNNRP in 2019 which showed that 129 (37.4%), Shashemene which was 54 (36.5%), and Bole sub-city Addis Ababa revealed that 18 (21.4%) [8, 11, 17]. The discrepancy might be that, in the current study, a high number of women received advice from a health professional about modern contraceptives., Most of the previous studies used a small sample size that may affect the prevalence; in addition, the current study included women in only one city with a large sample size, unlike the other studies in North-West with two cities, Amhara Regional State Zonal Towns and SNNRP.

This study showed that injectables, implants, and pills are the most commonly used modern contraceptives. This is similar to the studies conducted in Northwest Ethiopia (Gondar and Bahir Dar cities) and Bahir Dar [8, 17]. This may be because they are the most familiar and commonly used contraceptive methods at the national level, which leads them to recall and used it.

In this study, fear of side effects was the main reason for not currently using modern contraceptives. The qualitative result, which was the main reason for not using modern contraceptives, also supported this fear of its side effects. This is in line with the quantitative finding of a study conducted in Northwest Ethiopia (Gondar and Bahir Dar cities) [8]. This may be because of hearsay from friends or others, overestimation of the health risk of the side effects of modern contraceptives as its normal has a minimal side effect, while it would have rarely adverse side effects.

Sociodemographic characteristics significantly associated with street women aged 25–34 years were 5.5 times more likely to utilize modern contraceptives than those aged 15–24 years. A study conducted in Bahir Dar supports this finding [8]. This might be because those 25–34 years old street women are more likely to give birth, and they are more sexually active, which may lead to the use of modern contraceptives.

In this study, women who discussed modern contraceptives with a health professional were 14.3 times more likely to utilize modern contraceptives than their counterparts. These findings support the findings reported in Bahir Dar Town [17]. An explanation might be that as the participants receive counseling from health professionals, they can get the chance to understand the modern contraceptive methods, know the benefits of MCs, and about the side effects and misconceptions that can lead them to use them.

The odds of the utilization of street women toward modern contraceptives among those women discussed with their sexual partners about the utilization of MCs were 6.2 times more likely to utilize it than their counterparts. This finding is consistent with a study conducted in the Amhara Regional State Zonal Towns [19]. This could be because discussion might increase awareness about the value of contraception and initiate the study participants to use contraceptives.

Street women who faced sexual assault or rape in street life were 5.4 times more likely to currently utilize modern contraceptives than their counterparts. This finding is in line with a study conducted in the northwest (Gondar and Bahir Dar cities) [8]. This is because, as they face many problems, they are alert to care for themselves for the complications of unplanned and unwanted pregnancy because of unprotected sexual intercourse, which results in poor outcomes of abortion. This has led to the use of modern contraceptives.

In this study, the time taken to arrive at a nearby health facility on the foot was another predictor variable. Women who arrived at a nearby health facility on the foot within 30 min were 9.2 times more likely to utilize modern contraceptives than those who took more than 30 min. Other studies did not perform an association or were not associated. The justification might be that distance obviously can affect the seek-care of health behavior, as they are low-socioeconomic and may lack transportation costs to reach health facilities to get family planning services.

In our study, street women who had a history of pregnancy after joining the street were 2.7 times more likely to utilize MCs than their counterparts. This finding is consistent with a study conducted in the Amhara Regional State Zonal Towns in 2019 [19]. A possible reason might be that they have the chance to have desired children or that they may suffer from the complication of pregnancy at this time, which initiates them to utilize modern contraceptives.

This study has some limitations: as the participant’s reports of some sensitive variables such as sexual activity, abortion, and rape could be affected by social desirability bias, and they might have said that they may hide the truth. As we took all censuses of reproductive age street women, we faced some difficulties, such as the difficulty of searching the streets, and some women did not know their exact age. We faced an approximately 10% non-response rate due to participants’ refusal and incomplete responses.

## Conclusion

The proportion of street women currently using modern contraceptives is low. Factors associated with the utilization of modern contraceptives included age, arrival at a nearby health facility on foot, discussion about MCs with health professionals, discussion with sexual partners about the utilization of MCs, history of pregnancy after joining the street, desire time to have a child in the future, and history of rape in the street. Fear of side effects, misperceptions and myths, and desire to have a child are the main identified themes for the key barriers to using modern contraceptives. Besides, the above issues better to develop different strategies that can approach and increase the awareness and knowledge about modern contraceptives different methods, use and service availability, and accessibility among street women. Health professionals and health extension workers should give health education that integrates well-organized street women’s groups and community health extension workers that may help reduce women's fear of potential side effects. Additionally, to correct the misperceptions and myths about modern contraceptives too.

## Data Availability

The datasets used and/or analyzed are available from the corresponding author on reasonable request.

## Abbreviations

AOR: Adjusted odds ratio
CI: Confidence interval
IUCD: Intrauterine contraceptive device
MCs: Modern contraceptives
SD: Standard deviation
WHO: World health organization
